# Assessing the Photocatalytic Degradation of Fluoroquinolone Norfloxacin by Mn:ZnS Quantum Dots: Kinetic Study, Degradation Pathway and Influencing Factors

**DOI:** 10.3390/nano10050964

**Published:** 2020-05-18

**Authors:** Jyoti Patel, Ajaya K. Singh, Sónia. A. C. Carabineiro

**Affiliations:** 1 Department of Chemistry, Govt. V. Y. T. Post Graduate Autonomous College, Durg, Chhattisgarh 491001, India; jyotibhilai17@gmail.com; 2 LAQV-REQUIMTE, Department of Chemistry, NOVA School of Science and Technology, Universidade NOVA de Lisboa, 2829-516 Caparica, Portugal; sonia.carabineiro@fct.unl.pt

**Keywords:** quantum dots, Mn-doped ZnS, optical properties, photocatalysis, degradation

## Abstract

Norfloxacin (NOFX), a broadly used fluoroquinolone antibiotic, has been a subject of great concern in the past few years due to its undesirable effect on human beings and aquatic ecosystems. In this study, novel Mn doped ZnS (Mn:ZnS) quantum dots (QDs) were prepared through a facile chemical precipitation method and used as photocatalysts for NOFX degradation. Prior to photodegradation experiments, morphological and optical parameters of the QDs were examined through transmission electron microscopy, scanning electron microscopy, energy dispersive X-ray analysis, Fourier transform infrared spectroscopy, ultraviolet-visible spectroscopy, fluorescence spectroscopy, Brunauer–Emmett–Teller analysis, and differential thermal and thermogravimetric analyses. Mn:ZnS QDs exhibited excellent properties of photodegradation, not only under UV irradiation but also in sunlight, which induced NOFX to photodegrade. The utmost photodegradation efficiency was obtained under optimal conditions (25 mL of NOFX, 15 mg/L, pH 10, 60 min UV irradiation, 60 mgs QDs), adopting first order kinetics. In addition, hydroxyl radicals produced by the conduction band electrons were found to be the primary reason dominating the transformation of NOFX in basic conditions, while holes, oxygen atoms, as well as the doped metal (Mn) enhanced the degradation. The QDs showed excellent reusability and stability in four repeated cycles. Finally, four different pathways were predicted, derived from the identified intermediates, with piperazinyl ring transformation being the primary one. It is expected that the synthesized Mn:ZnS QDs could be utilized as efficient photocatalytic materials for energy conversion and ecological remediation.

## 1. Introduction

In recent years, there has been considerable worldwide expansion in the occurrence, behaviour, and fate of pharmaceutically active compounds used for the treatment of infectious diseases and for enhancing agricultural production [1]. Due to their widespread use and incomplete biodegradability, partial removal of these antibiotics is accomplished in usual wastewater treatment plants and quite large quantities are deliberately discharged into the environment. Consequently, due to the lack of effective treatments of antibiotic wastewater, these can be found in surface waters, causing unfavorable effects on aquatic and terrestrial organisms [2]. In biological treatment systems, fluoroquinolones (FQs) are hardly biodegradable due to their noxious consequences on microbial action. Moreover, the relentless contact of FQs with bacterial colonies produces a resisting influence against these antibiotics. A very small amount of antibiotic left-overs in the environment can cause eutrophication in ecosystems and infectivity in foodstuffs and drinking water sources [3,4,5]. A number of treatment processes dealing with antibiotic residuals have extensively been explored, such as electrolysis [6], microbial degradation [7], biodegradation [8], nanofiltration [9,10], activated carbon adsorption [11,12], and ozonation [13]. Those procedures showed different degrees of success in removing low concentrations of many organic compounds. However, treatments of the polluted wastewaters using these techniques are generally critical, ineffective, costly and lead to the production of derivatives, in addition to secondary waste products [14]. Hence, it is crucial to explore novel methods for wastewater management [15]. 

Photocatalytic degradation by semiconductors as photocatalysts is regarded to be an efficient means of treatment for antibiotic left-overs owing to its incomparable superiority, being ecologically friendly, highly efficient and with good stability [16,17,18]. Recently, quantum dots (QDs) aided photocatalytic degradation has been established as a competent and green method for exclusion of resistant organic pollutants from water [19,20]. The use of semiconductor materials, as a means for degrading the pollutants present in the environment under UV light has been broadly investigated in the last few years. Among the nano-scale materials, Zinc-containing QDs might be environmentally friendly, except at very high amounts, when they become noxious to living beings [21]. Also, zinc sulfide (ZnS) QDs are not poisonous, hence, they are the most popular preference for photocatalytic and biomedical uses [21,22]. Recently, ZnS, have been extensively inspected as novel semiconductors, in the field of photocatalysis, sensing and imaging, given their important properties, like visible-light response, suitable band positions, higher chemical and thermal stability, non-toxic and economical production [23,24,25]. However, reduced activity in visible light and the combination of photogenerated electron and hole pairs obstructs the large scale utilizations of these photocatalysts [26]. The modification with transition metal ion doping is highly important owing to lower noxious content, aqueous stability, improved surface area, economically feasible, biocompatible, and chemically inert character of QDs [27,28,29].

Doping is widely used as an efficient method to tune energy levels, surface states, as well as the structural, optical, magnetic and spintronic property of semiconductors [30,31]. Dopants play a vital role in semiconductor devices, which encouraged the study of the properties and possible applications of these semiconductor QDs, doped with intended impurities [32]. With their pioneering efforts, Bhargava et al. proposed, for the first time, this strategy of adding metal impurities at some stage of the nanoparticle synthesis to obtain doped QDs with modified optoelectronic and magnetic behaviour [33]. Doping ZnS nanoparticles with Mn^2+^ ions leads to luminescence enhancement and reduces their lifetimes, compared to bulk [34,35], along with lower toxicity, larger Stokes shifts, avoiding self-absorption, and improvement of thermal and environmental stability [36,37]. This is attributed to the interaction between the sp electron-hole (e^−^‒h^+^) of the host ZnS and the 3d e^−^ of Mn^2+^ions [34]. The energy from absorbed photons can be transmitted effectively to the Mn^2+^ ions, rapidly confining the excitation and restraining the undesirable reactions at the surface of the nanocrystal [38]. Furthermore, engineering of surface ligands in doped QDs improves their properties compared to the bare analogues, providing stability and solubility in biological environments [39,40].

Herein, we synthesized Mn doped ZnS (Mn:ZnS) QDs by introducing cetylpyridinium chloride (CPC) as capping agent on the surface of QDs for photoenhanced degradation of norfloxacin (NOFX). The modification of QDs with Mn doping efficiently augmented the absorption of light, improved the partition and transport of photogenerated electrons and reduced the electron–hole recombination. As-prepared Mn:ZnS QDs displayed good photocatalytic performance, stability, and reusability for photocatalytic applications.

## 2. Experimental

### 2.1. Materials and Apparatus

All the chemicals employed for syntheses were of analytical grade and did not need any further purification. Zinc acetate (Zn(CH_3_COO)_2_·2H_2_O), sodium sulphide (Na_2_S·9H_2_O), manganese carbonate (MnCO_3_) and cetylpyridinium chloride(C_21_H_38_ClN) of analytical grades were used in this research to prepare the ZnS:Mn^2+^ nanocrystal and were purchased from Merck (Mumbai, India). The laboratory reagent grade drug NOFX (Table 1) was supplied by Sigma-Aldrich (Bangalore, India). The pH values of solutions were adjusted using an aqueous solution of HCl and NaOH (Merck, Mumbai, India). Drug solutions with various concentrations were made by dilution of the stock solution, using double distillate water.

The nanoparticles morphology was studied using a JEOL-JEM 2100 transmission electron microscope (Cochin, India) and a Supra 55 Zeiss field-emission scanning electron microscope (Cochin, India). Fourier transform infrared (FTIR) spectra were obtained with Thermo Nicolet Avatar 370 (Cochin, India). The optical properties were studied using the absorbance spectrum recorded on a spectrophotometer Cary Win UV. The fluorescence spectrum was recorded using a JY Fluorolog-3-11 fluorescence spectrometer (Mumbai, India). N_2_ gas adsorption-desorption analysis was carried out using a Belsorp mini II (BEL Japan Inc, Osaka, Japan) at −196 °C (using liquid nitrogen). Surface areas were measured by the Brunauer–Emmett–Teller (BET) method, and the pore size distribution and volume using the Barrett–Joyner–Halenda (BJH) model. A systronic pH-meter was used for measuring the pH of sample solutions. For photocatalysis, the UV irradiations were carried out, using a mercury lamp of 40 W/m^2^ (Osram, Munich, Germany) with emission at 254 nm wavelength. Mass spectrometry was performed by Agilent 1290 Infinity UHPLC system (Agilent Technologies, Santa Clara, CA, USA).

### 2.2. Synthesis of Mn:ZnS QDs

Mn:ZnS QDs were synthesized by the chemical precipitation method [41,42]. In brief, aqueous solutions of sodium sulphide, zinc acetate and manganese carbonate were prepared. 0.5 M solutions of Zn(CH_3_COO)_2_·H_2_O and Na_2_S were employed as precursors. Then, 29.75 mL of a 0.01 M MnCO_3_ solution were added to 49.50 mL of a solution of (Zn(CH_3_COO)_2_·H_2_O) for 1% Mn^2+^ doping. After that, cetylpyridinium chloride (1.0 At.wt.%) was added as a capping agent. Then, the Na_2_S solution was added dropwise. A white precipitate appeared soon. The stirring continued for 15 min. The reaction was refluxed at 60 °C and then the solution was centrifuged at 5000 rpm for 4–5 min. The precipitate was then filtered through Whattman filter paper followed by washing to eliminate any adhered impurities.

### 2.3. QDs Based Photocatalytic Experiments for NOFX Degradation

The photocatalytic experiments were conducted using Mn:ZnS QDs to degrade NOFX in aqueous media. A stock solution of NOFX (100 ppm in water) was prepared for photodegradation. In order to optimize the nanocatalyst amount for drug degradation, solutions of different concentrations were taken and their pH was adjusted for the required values. The reaction solutions were prepared by the addition of QDs into each conical containing drug solution. The conduct tests were carried out in a batch reactor at constant temperature. The reaction temperature was maintained at 29 ± 1 °C. A 40 W/m^2^ mercury lamp (Osram) was employed as the ultraviolet (UV) light source (λ = 254 nm) during the degradation experiment. A suitable quantity of catalyst (0.01–0.08 g/L dose) was suspended into 25 mL of the NOFX solution with constant stirring. The solution was kept in the dark for 30 min before light illumination to accomplish adsorption-desorption equilibrium. After that, the lamp was switched on, and that moment was considered as “time zero” for the photocatalysis reaction study.

The solutions were irradiated by means of a UV lamp to provide energy. The distance between the solution and the UV light was set to 10 cm for the experiments. At defined time intervals, sample aliquots were taken from the solution, using a syringe, and the drug present was analyzed by spectrophotometry, after centrifugation. The decrease in absorbance (at λ_max_ = 275 nm) for drug samples after irradiation, for a given time interval, displayed the degradation rate, and thus the efficiency of the drug destruction and photocatalytic activity of QDs.

The reactive species capture study was also done in parallel to the photocatalysis experiments. This study was executed by addition of 0.01 M of various scavengers, for example, formic acid (HCOOH), potassium iodide (NaN_3_), sodium chloride (NaCl) and sodium azide (NaN_3_) before adding the photocatalyst into the NOFX solution. The percentage (%) of degradation was calculated from the following equation:

% Degradation = (1−C/C_0_) × 100 (1)

where C_0_ is the initial NOFX concentration and C is the concentration of NOFX solution, at time t after the UV light exposure.

## 3. Results and Discussion

### 3.1. Characterization of QDs

**Figure 1:**
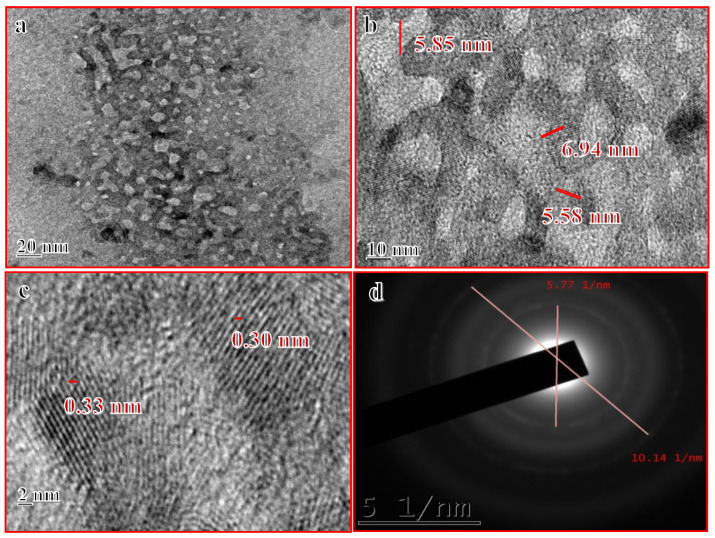
(**a**–**d**) HRTEM images of Mn:ZnSQDs.

After synthesis, the characterization of the Mn:ZnS QDs was accomplished by various techniques, to obtain information about the morphology of the prepared nanocatalysts. Figure 1a–d shows high resolution transmission electron microscopy (HRTEM) images of Mn:ZnS QDs. From the TEM micrographs, it is clearly observed that particles are nearly monodispersed with an average particle size of 6 nm.

**Figure 2:**
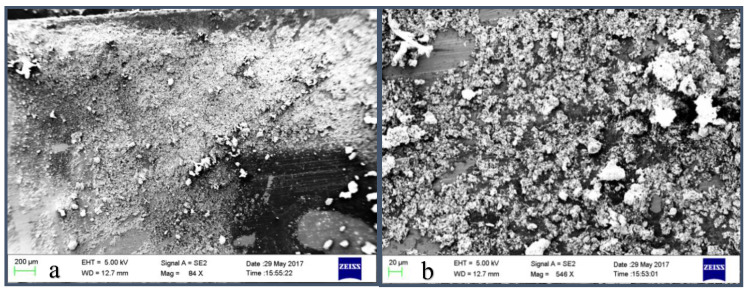
(**a**,**b**) SEM images of Mn:ZnS QDs.

The surface morphology of Mn:ZnS QDs samples was also studied using scanning electron microscopy (SEM). Figure 2 depicts the SEM images of the prepared nanocrystalline samples. The micrographs show that the synthesis method generates crystalline agglomerated nanoparticles of ZnS. The morphology reveals that as-synthesised Mn:ZnS QDs clusters are formed by prime building units of diverse orientations. Also there is a random aggregation between small particles, which leads to the development of irregular-shaped crystallites [43]. At lower magnification (Figure 2a) the micrograph shows un-homogeneities regarding particle size distribution. The higher magnification image (Figure 2b) clearly reveals that large particles are, in fact, agglomerates of the smaller sized particles.

**Figure 3:**
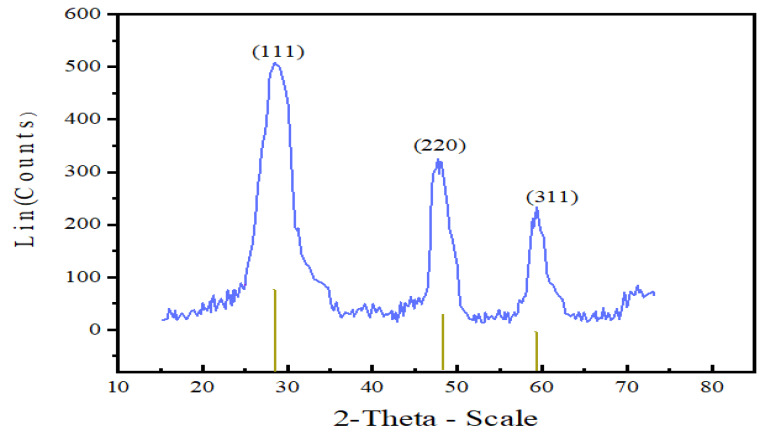
XRD diffractogram of Mn:ZnS QDs.

The XRD diffractogram depicted in Figure 3 shows that as prepared Mn:ZnS nanoparticles are crystalline and pure. The nanocrystals have less lattice planes than the bulk, which causes broadening of the peaks attributed to small particle effect [44]. Pure zinc blended crystal structure with the three broad peaks corresponding to the (111), (220) and (311) planes with reflections at 2θ = 28.4°, 48.3°, and 59.3° are observed. These results are in conformity with other experimental studies [45,46,47].

**Figure 4:**
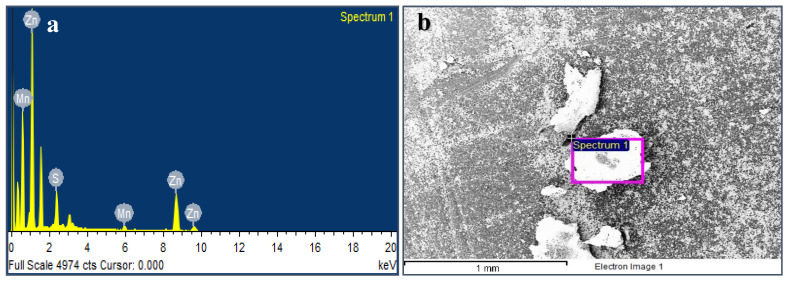
SEM image of Mn:ZnS QDs (**a**) with respective EDS pattern (**b**).

The energy dispersive X-ray analysis (EDS) revealed the elemental composition of the samples. Figure 4a shows the EDS of the average grains of Mn:ZnS QDs sample (Figure 4b). The analysis revealed the presence of 83.92 wt.% Zn, 13.32 wt.% S, and 2.77 wt.% Mn in the samples. Zn is present at 1.1, 8.7, and 9.6 keV, Mn at 0.5 and 6 keV, and S is present at 2.4 keV in the Figure 4b. Some more peaks are visible at 0.3, 1.6, and 3.1 keV, which are artificially (the so-called escape peaks) developed when the X-rays produced by fluorescence escapes the detector during analysis, as referred by the instrument supplier.

**Figure 5:**
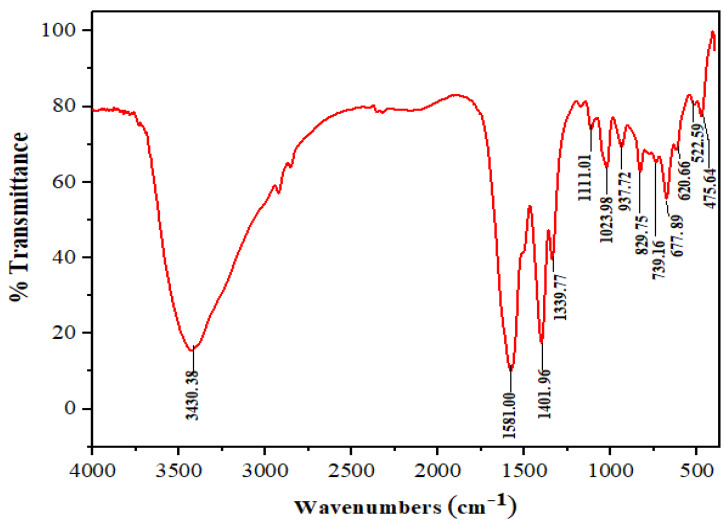
FTIR analysis of Mn:ZnS QDs.

The FTIR spectrum of Mn:ZnS QDs is shown in Figure 5. There are various features in the range of 500–4000 cm^−1^ associated with different functional groups [48,49]. The broad band at 3430 cm^−1^ is due to primary amine, N–H stretching. The bands at 2840 and 2930 cm^−1^ are due to a weak C–H stretching band. In the spectrum, the region between 1600–1300 cm^−1^ generally reveals the ring stretching vibrations. The absorption involves stretching and contraction of all the bonds in the ring and interaction between these stretching modes. Thus, the strong absorption peaks between 1401 cm^−1^ and 1581 cm^−1^ are due to aromatic C=C and C=N stretch in the ring. Bands corresponding to 1339 cm^−1^ indicate the presence of –CH_2_ group. The peaks between 730–830 cm^−1^ are ascribed to C–H deforming vibration. Some other small peaks are also present in the spectrum, however not discussed here. Thus, the FTIR study strongly supports the formation of CPC capped Mn:ZnS QDs.

**Figure 6:**
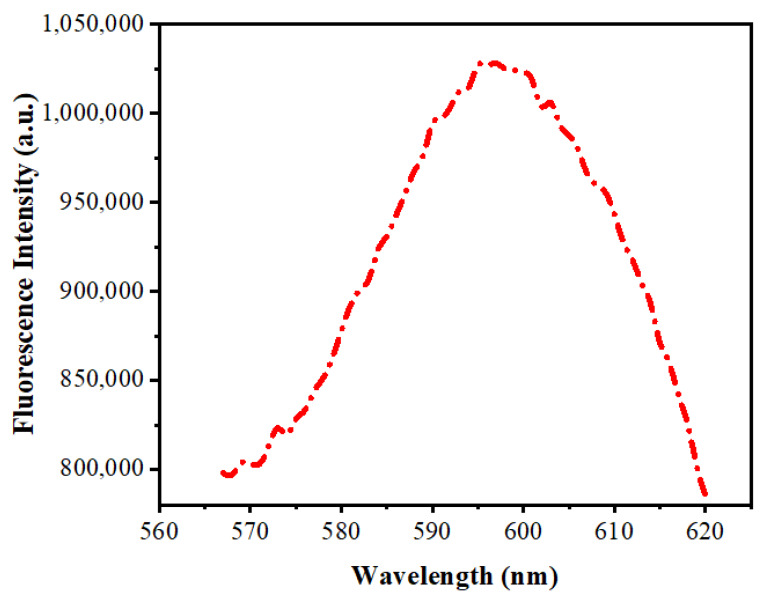
Fluorescence spectra of Mn:ZnS QDs.

Figure 6 depicts fluorescence spectra of Mn:ZnS QDs. There is a yellow-orange emission at about 598 nm. The ^4^T_1_–^6^A_1_ transition in the 3d shell of Mn^2+^ ions is the reason for this emission. It is due to the proficient transfer of energy from the ZnS to the doped Mn^2+^ and is assisted by the mixed electronic states [50]. Whilst the Mn^2+^ ion is incorporated in the lattice of ZnS, the cationic sites get substituted, and there occurs a mixing of s–p electrons of ZnS and the d electrons of doped Mn^2+^, making the ^4^T_1_–^6^A_1_ forbidden transition partly possible, which leads to the yellow-orange emission of the Mn^2+^ ions. 

**Figure 7:**
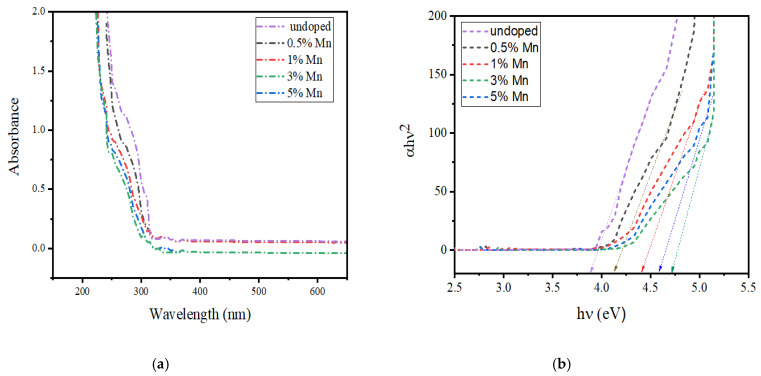
(**a**) UV-visible absorption spectra of Mn:ZnS QDs. (**b**) Band gap energy curve of Mn:ZnS QDs.

Figure 7a shows the ultraviolet-visible (UV-vis) spectra of pure ZnS and Mn:ZnS QDs. The absorption band of the QDs can be found between 250 to 290 nm, but in case of bulk ZnS, the band is observed near 350 nm with an energy-gap of 3.68 eV [51]. Figure 7b shows the band gap energy curve of pure ZnS and Mn:ZnS QDs calculated from the Tauc’s relation [52]. The direct optical energy gap value (E_g_) obtained for pure ZnS is 3.88 and for Mn doped ZnS QDs it is found to be 4.12, 4.39, 4.6, and 4.5 for 0.5%, 1%, 3%, and 5%Mn doping. The observed increase in E_g_ value, as well as the blue shift of the absorption spectra, might be caused by the decline in the size of the particles, due to the quantum size confinement [53,54]. A slight decrease in the E_g_ value is found above 3% Mn doping which indicates that the particle size increases with a further increase in Mn concentration [55].

**Figure 8:**
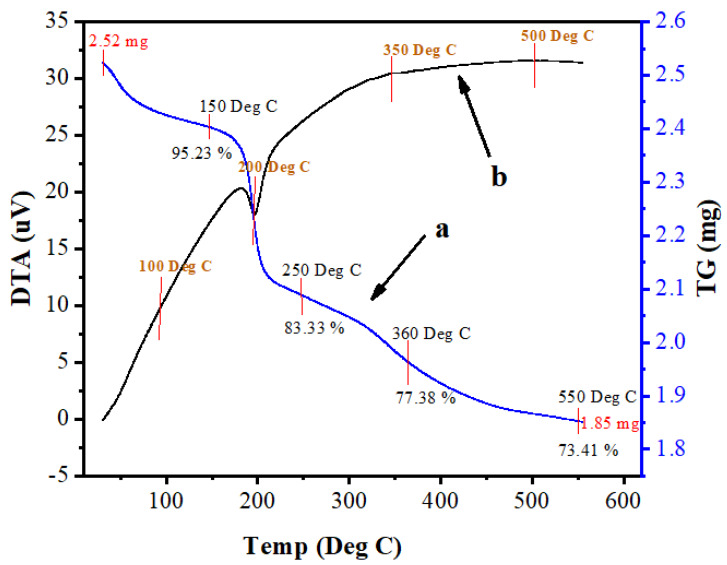
Thermogravimetric analysis (TGA, **a**) and differential thermal analysis (DTA, **b**) of Mn:ZnS QDs.

The thermal decomposition, stability and temperature of phase formation study of Mn:ZnS QDs was carried out by thermal analyses. Differential thermal analysis (DTA) and thermogravimetric analysis (TGA) were conducted to evaluate the thermal behaviour of the complexes. Data was collected from room temperature up to 700 °C, with a heating rate of 10 °C/min. Figure 8a,b shows the TGA and DTA curves of Mn:ZnS QDs. The endothermic peak near 200 °C is due to the loss of physically adsorbed water. In the TGA plot, various stages of weight loss are observed. The first weight loss up to 250 °C (16.67%) is related to physically adsorbed water molecules. The second weight loss (5.95%), between 260 and 360 °C, is associated with the degradation of organic groups coming from the precursor particles. The third (3.97%) deals with the release of Mn ions, as well as decomposition of residual S ions from the sample. Curve A shows that the analysis started with 2.52 mg sample weight, and after heating to 600 °C, the sample weight was 1.85 mg. Thus, the weight loss percentage up to 700 °C was 26.5%. The DTA curve at Figure 8b shows an exothermic process up to 460 °C, which might be due to a change in crystallinity or a phase change in the material.

**Figure 9:**
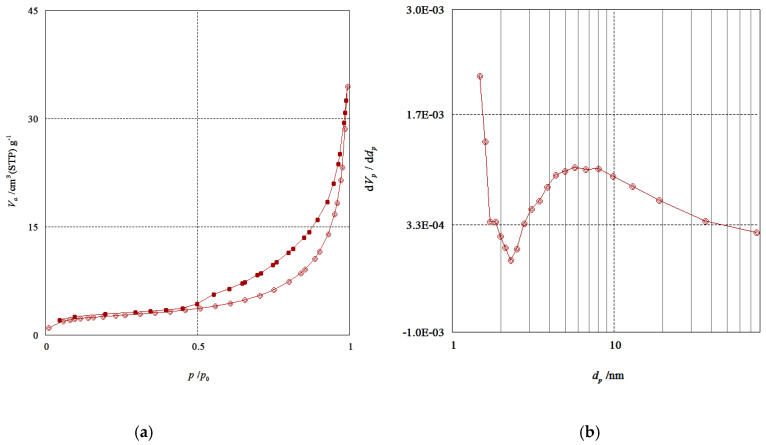
(**a**) N_2_ adsorption-desorption isotherms of Mn:ZnS QDs. (**b**) Pore size distribution calculated from the adsorption branch.

Nitrogen adsorption-desorption experiments at −196 °C were performed to confirm the porous structure of QDs. Figure 9a shows a typical type IV isotherm curve with an adsorption hysteresis loop and Figure 9b shows the pore size distribution. The surface area value of QDs was calculated using the BET equation and the value obtained was 10 m^2^/g. The sample displayed an extensive allocation of pore sizes with an average diameter of 5.7 nm (calculated from the BJH method) and the total pore volume was 0.05 cm^3^/gm. 

### 3.2. Photocatalytic Degradation of NOFX Using Mn:ZnSQDs

**Figure 10:**
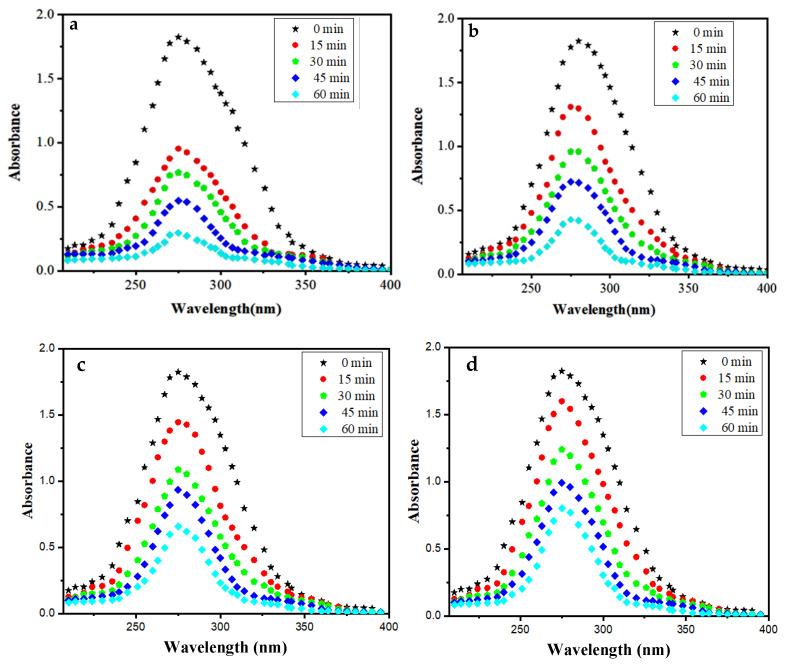
Absorption spectra of NOFX (15 mg/L) under optimal conditions (25 mL of SDB, pH 10, 60 min, 60 mgs QDs in the presence of (**a**) Mn:ZnS QDs in UV light; (**b**) Pure ZnS QDs in UV light (**c**) Mn:ZnS QDs in sunlight (**d**) Pure ZnS QDs in sunlight).

In order to investigate the photocatalytic behaviour of the synthesized Mn:ZnS QDs, a sequence of photocatalytic experiments for the degradation of drug NOFX was carried out under UV light radiation. The UV-vis absorption spectra of NOFX drug (15 mg/L) were recorded with wavelength of 200–400 nm as a function of illumination time (Figure 10a). From the absorption spectra, the absorbance of NOFX is gradually decreased with irradiation time. The effect of various process parameters, such as solution pH, catalyst dose and initial drug quantity on the degradation efficiency of NOFX was assessed by varying pH (2–11), catalyst dose (20–90 mgs), maintaining the fixed drug concentration (15 mg/L). Figure 10a shows that about 86% NOFX (15 mg/L, pH 10) was decomposed in the presence of the photocatalyst Mn:ZnS QDs (60 mgs) after 60 min of UV light exposure. The decrease of absorption spectra and, hence, absorbance of samples at λ_max_ = 275 nm indicates the degradation of NOFX drug in the applied situations. As a result, the decrease in the absorbance of samples due to decrease of drug concentration is traced for the measurement of degradation rate.

The photocatalytic effect of Mn:ZnS and pure ZnS QDs on the degradation process of drug NOFX can be observed in the corresponding absorption spectra in the presence of UV light (Figure 10a,b) and sunlight(Figure 10c,d). Thus, NOFX could be well degraded with different light sources, viz., UV irradiation and sunlight. It was found that Mn:ZnS QDs exhibited excellent properties of photodegradation not only under UV irradiation but also under sunlight, which caused the NOFX to photodegrade.

**Figure 11:**
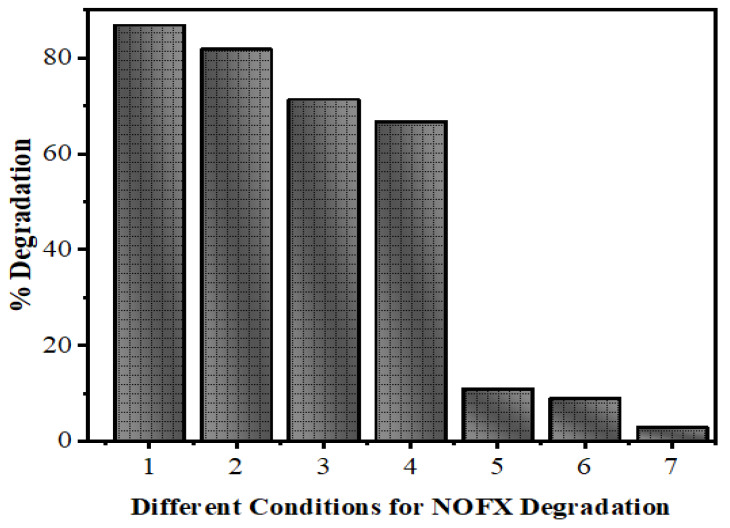
Efficiency of ZnS QDs for NOFX (15 mg/L) obtained in different conditions under optimal parameters (25 mL of SDB, pH 10, 60 min irradiation, 60 mgs QDs).(1) Mn:ZnS + UV, (2) Pure ZnS + UV, (3) Mn:ZnS + Sunlight, (4) Pure ZnS + Sunlight, (5) UV light (no catalyst), (6) ZnS QDs, (7) Blank (without light and catalyst).

In Figure 11, the evaluation of photodegradation of NOFX with concentration of 15.0 mg/L is shown for pure ZnS and Mn:ZnS QDs with sunlight, UV light and other conditions with catalyst dosage 60 mgs for 60 min. The photodegradation efficiency for NOFX obtained in different conditions followed the order: 

Blank (experiment performed without light and QDs) < ZnS QDs < UV light (no QDs) < pure ZnS in sunlight < Mn:ZnS in sunlight < pure ZnS in UV light < Mn:ZnS in UV light. 

#### 3.2.1. Effect of the Initial pH of NOFX Solution

**Figure 12:**
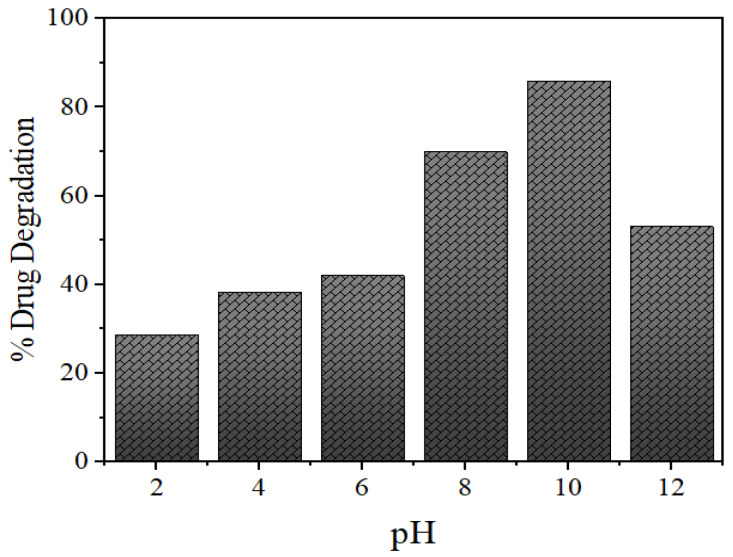
Effects of pH on photocatalytic degradation of NOFX in the presence of Mn:ZnSQDs under optimal conditions (25 mL of NOFX, 60 min irradiation, 60 mgs QDs).

The pH of the solution plays a significant role in the photocatalytic aqueous oxidation of organic compounds, since the pH value can influence various properties of the photocatalyst, for example, charge of the catalyst, size of the catalyst particles and the positions of valence and conductance bands. To get acquainted with the pH effect, degradation experiments were performed at varying pH values (2.0–12.0) with 25 mL of 15 mg/L initial NOFX concentration and 60 mgs QDs, at room temperature, for 60 min. Figure 12 shows the effect of pH values on the drug degradation over time.

As expected, the photocatalytic degradation of NOFX occurred faster at basic pH, compared to lower values of pH (Figure 12), similar to earlier experiments with the same drug [56,57]. The photocatalytic degradation increased with pH increase, but maximal adsorption and degradation were obtained at pH 10.5–11. A higher value of pH lead to a lower level of adsorption and degradation of the drug. In fact, the surface charges of NOFX and Mn:ZnS catalyst are the main aspects behind this behaviour.

For ZnS, the point of zero charge (pH_zpc_) value is 7–7.5, thus, the surface charge of the ZnS QDs is negatively charged above pH 7, whereas, it is positively charged at pH values below 7 [53]. NOFX has two ionizable functional groups: the 3-carboxyl group and N4 of piperazine moiety inducing two different ionization constants: p*K*a_1_ (pH = 6.34), due to deprotonation from the carboxyl moiety; and p*K*a_2_ (pH = 8.75), due to a protonation to N4 in the piperazinyl substituent. Thus, NOFX behaves as positively charged below pH 6.34, negatively above 8.75, and zwitterionic between these two pH values [58].

When the solution is acidic, NOFX and Mn:ZnS QDs are both managed by positive charge. The like charges show repulsive action, restricting the interaction of NOFX with the QDs surface, thus decreasing the rate of degradation. The attractive force between the deprotonated carboxyl moiety and the QDs forwards the degradation reaction [59]. Most competent and optimal NOFX degradation take place at the pH values lightly exceeding the pKa_2_, i.e., between 9.5–10.5. This may be attributable to a larger concentration of hydroxyl anions (OH^−^), which are the source of hydroxyl radicals during photocatalysis. The higher the OH^−^ ion concentration, the larger will be the generation of hydroxyl radicals (OH^•^) [60]. Above this pH, degradation efficiency decreases, possibly due to the repulsion between negative charges of NOFX and ZnS QDs. Thus, pH 10 was selected as optimal pH value for further studies.

#### 3.2.2. Effect of Catalyst Loading

**Figure 13:**
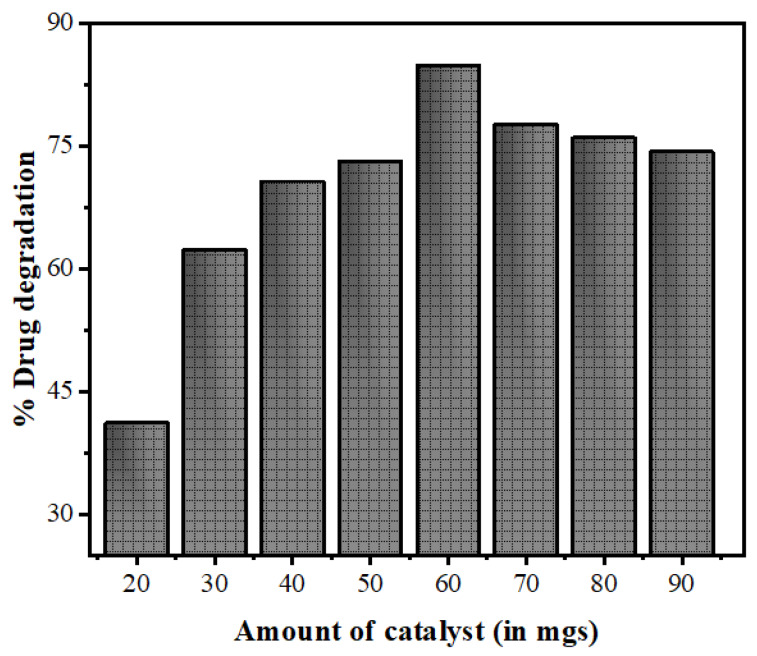
Effects of variation of the amount of catalyst Mn:ZnSQDs on the photocatalytic degradation of NOFX under optimal conditions (25 mL of drug, pH 10, 60 min irradiation).

The nanophotocatalyst dose is an important parameter in the photocatalytic degradation of pollutants as the amount of the catalyst directly controls the transformation rate. In the present study, the reactions were done by varying the catalyst amounts (ranging from 20 to 90 mgs) while keeping the drug concentration fixed at 15 mg/L. The degradation of NOFX increased from 41% to 84% as the catalyst amount increased from 20 to 60 mgs/25 mL, and decreased to 79% on further increasing the catalyst loading for 1 h reaction. The initial increase in NOFX degradation may possibly be due to an augmentation in the active sites accessibility as catalyst loading increased. However, a further addition of catalysts reduced light penetration (owing to light scattering from suspension), thus the volume of photoactive suspension decreased. The results of Figure 13 illustrate that 60 mgs/25 mL catalyst is the optimal value for maximal drug degradation. As a result, further experiments were made with this amount of catalyst loading.

#### 3.2.3. Effect of the Initial Concentration of Drug

**Figure 14:**
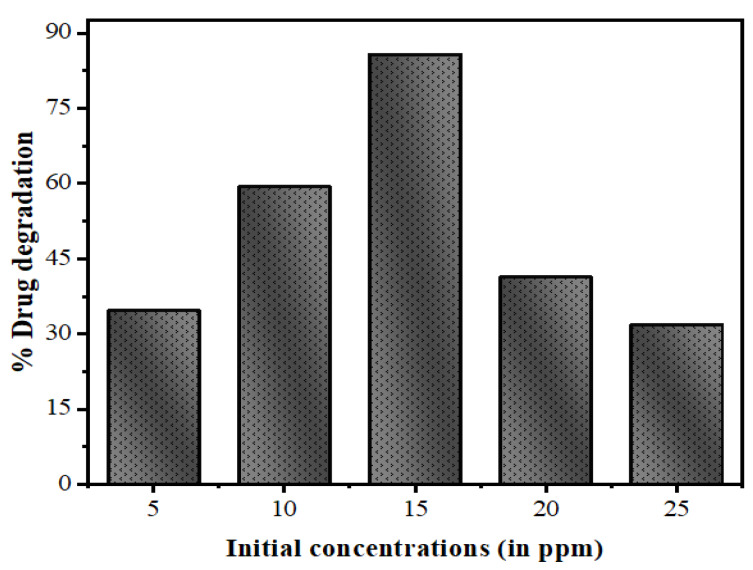
Effect of variation of the initial concentration of drug NOFX on the photocatalytic degradation under optimal conditions (25 mL of drug NOFX, pH 10, 60 min irradiation, 60 mgs QDs).

Treating the effluents present in the industrial and biological wastewater produced by natural and anthropogenic sources is a major challenge. Sometimes, industrial contaminants are chemically stable, and have inhibitory effects for biological treatment systems [61]. Thus, it becomes essential to develop methods for the removal of contaminants at high concentrations from the wastewater prior to releasing them into the environment. Therefore, in the present method, the photodegradation was investigated at different concentrations, including 5, 10, 15, 20, and 25 mg/L with a fixed quantity of photocatalysts, as shown in Figure 14. It can be observed that the degradation rate of drug decreases with the increasing drug concentration, showing elevated degradation efficiency for a low initial concentration.

This decline of efficiency with rising drug concentration is attributable to two reasons. As the initial amounts of drug increases, higher extent of drug molecules will be adsorbed over the surface of the QDs and the active sites will decrease. This leads to an increase in the occupied space of catalyst surface; resulting in reduced OH^•^ radicals generation. Moreover, higher drug concentration causes less number of photons reaching the catalyst surface. Thus, most of the light is prevented by the adsorbed drug molecules and the photoexcitation of catalyst QDs declines, causing the slow degradation rate detected in the later part of the experiment [62].

#### 3.2.4. Effect of Mn^2+^ Dopant Concentration

**Figure 15:**
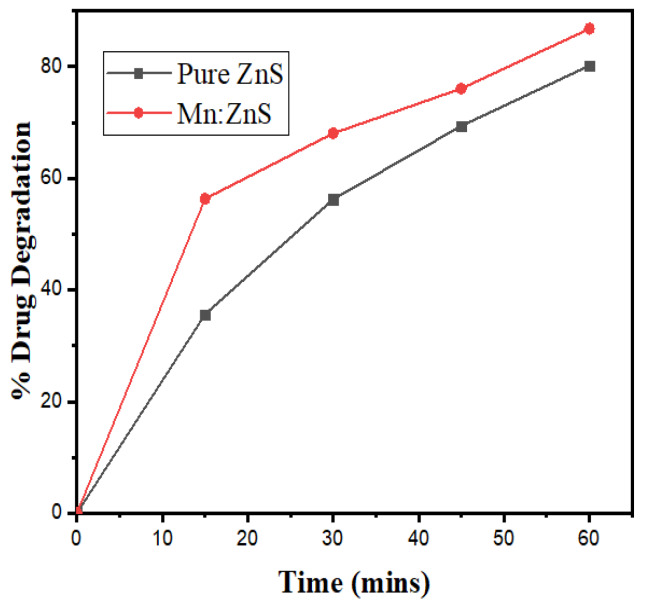
Effect of Mn^2+^doping on the percentage degradation of drug NOFX under optimal conditions (25 mL of drug NOFX, pH 10, 60 min irradiation, 60 mgs QDs).

In order to study the Mn^2+^ doping effect on the photodegradation efficiency of ZnS QDs, experiments were carried out under optimal conditions with different dopant amounts of Mn (0.5, 1.0, 3.0, 5.0% *w*/*w*). A brief comparison between the obtained degradation efficiency of NOFX in the presence of undoped and Mn doped ZnS QDs (Figure 15) indicates that the doping of Mn in the crystalline structure of ZnS, as an impurity, has a positive effect on the degradation efficiency through photocatalysis. The improvement found after doping compared to the undoped sample is 4.2%, which is close to the values found in literature 2–3% [21,23,63]. 

**Figure 16:**
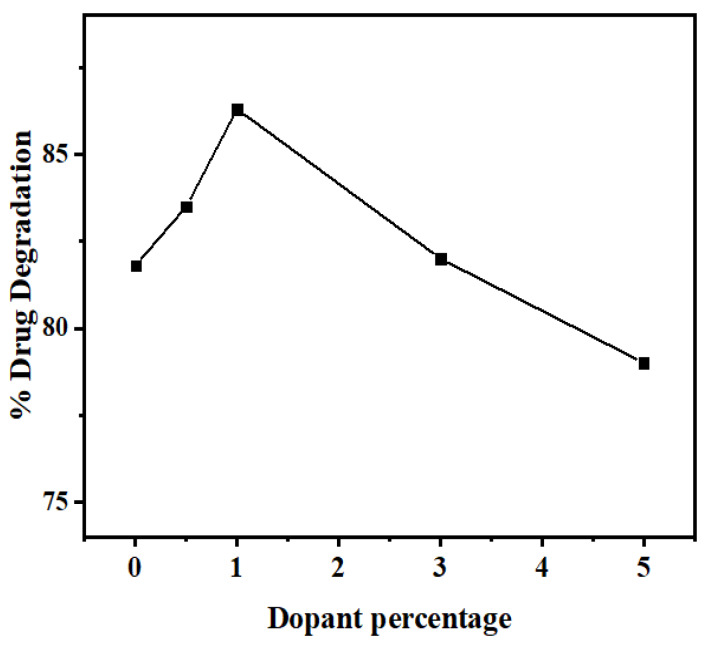
Effect of Mn^2+^dopant percentage on the photocatalytic degradation of drug NOFX under optimal conditions (25 mL of drug NOFX, pH 10, 60 min irradiation, 60 mgs QDs).

Nevertheless, a negative result in photocatalysis efficiency was observed for higher dopant concentrations. As seen in Figure 16, the photodegradation efficiencies of the QDs slightly increase from 0.0% to 1.0% and decline above 1% of the dopant concentration.

The changes observed upon the doping process might possibly be due to the variations in the crystallite size of the QDs, which may influence their photocatalytic performance [64]. Moreover, the dopants make the shallow trapping sites available for the charge carriers in the structure of the semiconductors. Consequently, the quantity and recombination rates of these charge carriers may vary by separating the arrival times of charge carriers at the surface [65,66]. At low dopant concentrations, Mn^2+^ ions act for h^+^ trapping only. However, at higher concentrations, the ions work as traps both for e^−^ and h^+^, leading to their recombination by quantum tunneling [64,67,68].

### 3.3. Kinetic Studies

The mechanism and the efficiency of the photodegradation of NOFX can be elucidated from the kinetic studies. They provide an indication for the effectiveness of the photocatalytic process. The studies were done at the optimal conditions for NOFX drug solution. The first order kinetic model was applicable and carried out using a linear fitting of ln C_0_/C_t_ versus *t*:

(2) $$\ln\frac{C_{0}}{C_{t}}=\mathrm{kt}$$

where C*_o_* and C*_t_* represent the concentration of NOFX at time 0 and time t, respectively, and k is the observed first order rate constant (min^−1^). 

**Figure 17:**
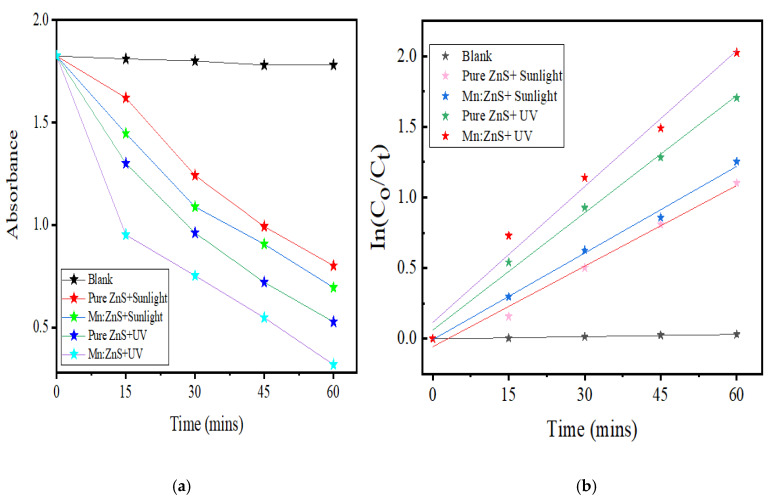
(**a**) Changes in the corresponding maximum wavelength of NOFX. (**b**) First order kinetic model fitting to the QDs-based photocatalytic degradation of NOFX data with Mn:ZnS QDs under optimal conditions (25 mL of 15 mg/L NOFX, pH 10, 60 min UV irradiation, 60 mgs QDs).

The NOFX degradation rates under sunlight and UV light were obtained from the plot slopes of Figure 17a,b. The R^2^ values show that the first-order model can be applied to the obtained experimental data. The values for the first-order kinetic data, together with rate constants (k), correlation coefficients (R^2^) and degradation efficiency for Mn:ZnS QDs for different conditions of photocatalysis are given in Table 2. The photocatalytic activity of synthesized pure ZnS and Mn:ZnS QDs under UV light and natural sunlight can be assessed by comparing the apparent rate constants. Thus, it can be seen that the photodegradation efficiencies of pure ZnS and Mn:ZnS QDs as catalysts under UV light illumination are higher than under natural sunlight irradiation.

### 3.4. Probable Photocatalytic Degradation Mechanism of the Photocatalyst 

The semiconductor photocatalyst can be excited when irradiated with photons of light equal or slightly higher than its band gap [63]. Thus, the possible mechanism for UV light assisted photodegradation of NOFX starts with the excitation of the semiconducting QDs, resulting in the formation of e^−^–h^+^ pair at the QDs surface, which are the charge carriers. These e^−^ and h^+^ can either recombine or drift to the surface giving rise to the photo-active centres [69]. Actually, the overall number of charge carriers (e^−^ and h^+^) at the photocatalyst surface determines the efficiency of degradation.

The h^+^ can indirectly or directly oxidize the organic matter (NOFX drug) due to its high oxidation potential. During indirect oxidation, the reactive hydroxide radicals (OH^•^) are produced due to the combination of h^+^ with H_2_O or with hydroxide anions [70], as shown in the following equations:

Qd + hv → Qd (e^−^ + h^+^), (3)

h^+^ + Drug → Oxidation of Drug, (4)

h^+^ + OH^−^ → OH^•^, (5)

h^+^ + H_2_O → H^+^ + OH (6)

This OH**^•^** is a strong oxidative (*E*◦ = +3.06 V) and non-selectively oxidizes drugs and other organic matter to mineral species partially or completely. Moreover, oxygen atoms as well as the doped metal at the surface work as a sink for e^−^ enhancing the e^−^−h^+^ separation. The conduction band e^−^ at the surface of ZnS could reduce molecular oxygen (O_2_) to superoxide anion (O_2_**^•^**^−^). The O_2_**^•^**^−^ subsequently reacts with H_2_O producing H_2_O_2_, which then generates OH^•^ radicals [71].

e^−^ + O_2_ → O_2_^•^^−^, (7)

O_2_^•^^−^ + H_2_O → H_2_O_2,_ (8)

H_2_O_2_ + e^−^ → 2OH^•^_,_ (9)

Depending upon the precise experimental situation, the h^+^, e^−^, OH^•^, O_2_**^•^**^−^ and O_2_ itself play significant roles in the photodegradation mechanism [72]. Consequently, the OH^•^ generated by e^−^ in the conduction band, in addition to various other stages, can cause organic matter mineralization:

HO^•^ + Drug molecules → Degradation of Drug, (10)

HO^•^ + O_2_^•^^−^ + Drug → CO_2_ + H_2_O + small less toxic species, (11)

In case of doping the catalyst with transition metal ions, the improvement of photocatalytic degradation is due to additional OH^•^ and O_2_**^•^**^−^ formation. Since doping provides a means to trap charge carriers (e^−^ and h^+^), extending their lifetimes, consequently, dopants increase the photocatalytic efficiency [73].

### 3.5. Role of Active Oxidation Species

**Figure 18:**
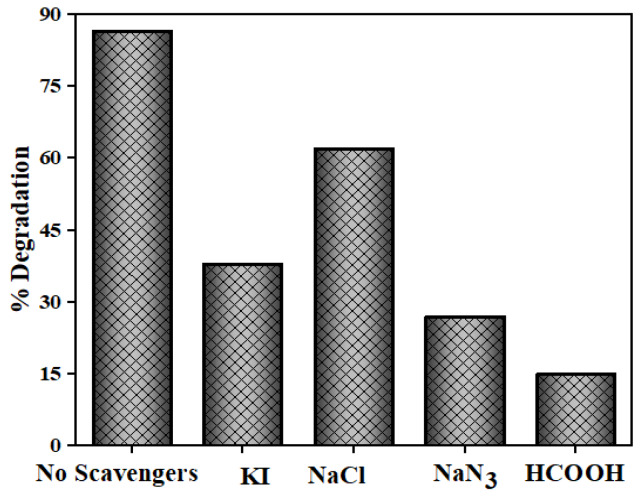
Photocatalytic degradation of NOFX with different scavengers.

The generation and functions of reactive species, like h^+^, O_2_^•─^, OH^•^ and ^1^O_2_ for NOFX degradation was studied by adding 0.01 M of appropriate scavengers of these species. Due to divergence in the energy gaps and phase compositions, there may be variance observed in the functions of active species for different photocatalysts. KI is known to be an efficient quencher for h^+^ and OH^•^s present at the QDs surface [74,75]. As illustrated in Figure 18, NaCl has been added as h^+^ quencher, HCOOH for e^−^ and NaN_3_ for ^1^O_2_ and OH^•^ quenching [75]. The scavengers were added prior to the addition of the photocatalyst. The highest NOFX degradation (86%) was observed in the absence of any scavenger. On the contrary, the hinderance of photodegradation efficiency was about 15%, 62% and 27% when HCOOH, NaCl and NaN_3_ were used as scavengers substantiating the crucial role of e^−^ and ^1^O_2_ in photocatalytic method. Also, the photodegradation efficiency turned down to 38% in the presence of KI, proving that the h^+^ and OH^•^s also take part in the transformation of NOFX. 

### 3.6. Identification of Transformation Products

**Figure 19:**
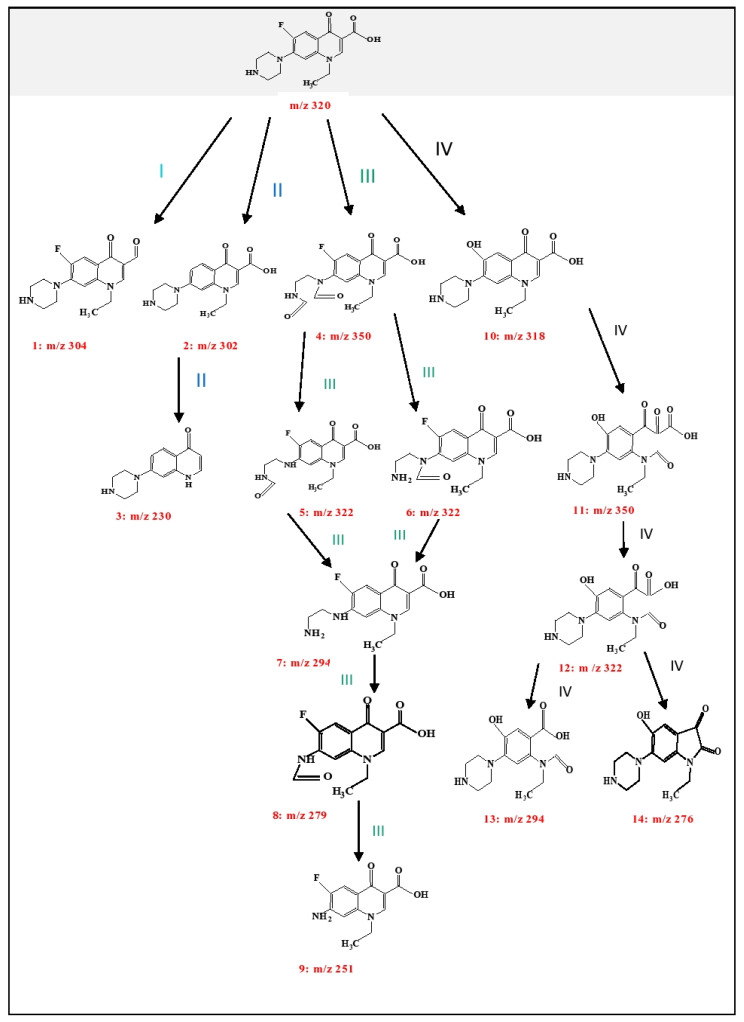
Probable photocatalytic degradation pathway of NOFX.

It is also important to be acquainted with the degradation products resulting from the Mn:ZnS QDs assisted photodegradation of NOFX, as some of the products may be more dangerous and unsafe to the environment. The possible degradation pathway of NOFX with Mn:ZnS QDs as photocatalyst, was conjectured by the results of mass spectrometry. The structures of the intermediates produced were tentatively recognized on the basis of the fragmentation patterns acquired from high resolution liquid chromatography mass spectrometry (HRLC MS) analysis and the existing information obtained from previous literature [76,77]. Primarily, the degradation initiates by the attack of radicals on piperazinyl and quinolone moieties. A total of 14 intermediates were identified in the degradation pathway described in Figure 19. The pathways for NOFX degradation were proposed based on the recognized intermediates.

In the current case, for pathway I, a dehydroxylation reaction takes place in NOFX molecule giving rise to structure **1** (m/z 304). In several cases, structure **1** further decomposes by losing a carbonyl group thereby producing its protonated form with m/z value 276 [78]. In second pathway, defluorination reaction occurs probably generating **2**, which is the protonated form with m/z 302. Finally, the **2** transmits to **3** (m/z 230) by decarboxylation and deethylation reaction [79,80,81].

As discussed earlier, the piperazinyl moiety in NOFX is further a dynamic group for radical attack. Pathway III is chiefly the obliteration of the piperazinyl group. Six intermediary structures were recognized at this stage via oxidation, ring-opening and elimination reactions [78,82]. The protonated forms occur at m/z 350, 322, 294, 279, and 251 respectively. Initially, NOFX gets oxidized, resulting in opening of the piperazine ring, generating **4**. Consequently, structure **4** loses its two ‒CO groups and is converted into **7** (through **5** or **6**), which is similar to Guo’s results [56]. Again, **7** gets oxidized, producing **8**, which loses a ‒CO group and is transformed into **9** [83]. Ultimately, the piperazinyl moiety of NOFX is entirely destroyed. Thus, cleavage of piperazine ring played an important role in the photocatalytic transformation of NOFX, which is also manifested through the preceding literature [56,83,84,85]. Pathway IV comprises the opening of quinolone substituent and the benzene moiety. On hydrolysis, the F in NOFX gets substituted with OH^•^ group, which forms **10** [80]. Liu et al. specified that the OH^•^ attacks the quinolone moieties in NOFX at the carbon–carbon double bond closest to the ‒COOH group [82]. Hence, the protonated products with m/z 350 (product **11**), followed by 322, 294, and 276 (products **12**, **13**, and **14**) are possibly expected to be generated. Thus, from the above discussions, NOFX could be decomposed into **3**, **9**, **13**, and **14**, which are easy to be mineralized further to small molecules like CO_2_, H_2_O, NO_3_^−^, and F^−^ on account of their lower stability [79].

The prepared Mn:ZnS QDs powder materials exhibit excellent degradation efficiency in comparison to other photocatalysts, due to the larger number of charge carriers caused by the addition of Mn as an impurity, and the differences in the arrival time of e^−^ and h^+^ at the photocatalyst surface. Therefore, it was found that TiO_2_, ZnO and some other nanocomposites (often used for degradation) are less efficient in degrading NOFX. Until now, the photocatalytic degradation of NOFX using Mn:ZnS QDs were not explored in literature. But other photocatalysts have been used for the degradation and mineralization in aqueous phase. 

Yang et al. prepared a series of anatase, rutile and brookite structures of TiO_2_ as photocatalysts enabling complete degradation of 100 µM L^−1^ NOFX in 240 min with a dose of 0.1 gm TiO_2_ [86]. According to Shankaraiah et al., about 90% of NOFX (150 mg/L) was accomplished under UV light after 180 min irradiation with 0.3 g TiO_2_. Also photodegradation experiments were done with TiO_2_-immobilized glass beads UV light where 78% degradation was achieved in 180 min with 10 gm beads [87]. Nekouei et al. prepared N-doped activated carbon-CuS nanocomposite for NOFX degradation assisted by visible light where 80% degradation of 60 mg/L NOFX was observed after 120 min light irradiation with 50 mg of nanocomposite [88]. Li et al. [89] developed a photocatalytic redox reaction system to improve the performance of photocatalytic fuel cell by combining it with persulfate (S_2_O_8_^2^^−^) using TiO_2_ nanorods photo anode leading to NOFX degradation from the surface of electrodes to the entire solution. From the results, it was found that 75% degradation of 30 mg/L NOFX was accomplished in 60 min. Zhang et al. prepared Zn_0.9_Fe_0.1_S/Ni-foam nanocomposite as photocatalysts, and degraded 95% of 5 mg/L NOFX in 120 min with 0.03 gm of the nanocomposite [90]. Shah et al. employed the as-synthesised Bi^3+^‒Fe^2+^ co-doped ZnO for NOFX degradation and attained 80% removal of 10 mg/L NOFX in 120 min using 1 g/L catalyst [91]. Jin et al. used N-doped TiO_2_ for NOFX degradation where 69.79% degradation of 12.5 mg/L NOFX was obtained after 30 min of visible light irradiation with 0.4 g/L of the photocatalyst [92].

### 3.7. Reusability of Photocatalyst for Degradation of NOFX

**Figure 20:**
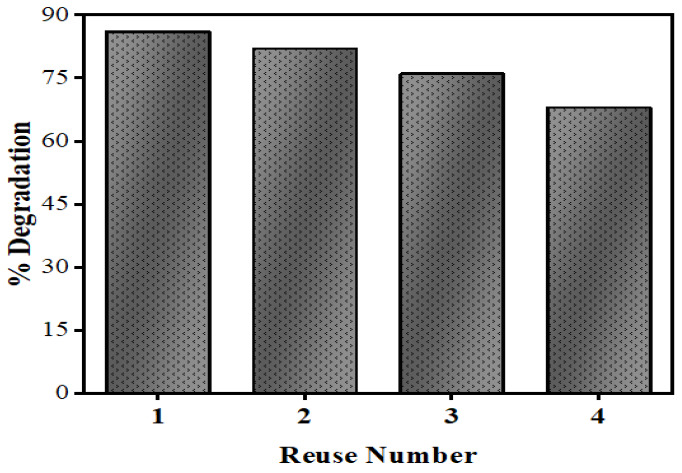
Reusability of Mn:ZnS QDs for NOFX degradation.

Figure 20 shows the recyclability of the Mn:ZnS QDs powder photocatalyst for the degradation of NOFX. After every run, the QDs were separated from the drug solution by centrifugation, washed with distilled water and dried to study the catalysts recyclability. Afterwards, the dried photocatalyst was redistributed in fresh NOFX solution for the degradation experiments under optimized conditions. The degradation efficiency decreases from 86% to 65% after the 3rd repeated cycle of photocatalysis, which may be attributed to the loss of a small amount of catalyst after every run.

## 4. Conclusions

In this study, Mn:ZnS QDs were prepared by a facile and rapid chemical precipitation method and the photocatalytic degradation of NOFX in the presence and absence of pure ZnS and Mn:ZnS QDs was investigated. In the photocatalytic degradation, the optimal parameters involved (substrate concentration, loading of catalyst, and solution pH) were identified. The synthesized Mn doped photocatalyst was much more efficient for the degradation of NOFX than the undoped material. This can be explained by the enhanced e^−^−h^+^ separation at the hetero interface, by the production of highly reactive radicals and by an enlarged active surface area. The photocatalytic drug removal process followed first-order kinetics. Thus, Mn:ZnS QDs mediated the efficient degradation of NOFX, suggesting that the process is highly efficient, very simple, and can potentially be used for the remediation of organic pollutants from waste waters.

## Figures and Tables

**Table 1:** Physicochemical characteristics of norfloxacin.

Name	Molecular Structure	M_w_ (g/mol)	λ_max_	Molecular Formula
Norfloxacin	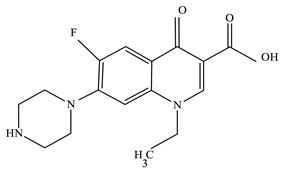	319.33	275 nm	C_16_H_18_FN_3_O_3_

**Table 2:** Parameters for the removal of NOFX by Mn:ZnS QDs.

Condition	Rate Constant k (min^−1^)	R^2^	DE (%)
Blank	5.0 × 10^‒4^	0.971	2.93
Pure ZnS + Sunlight	1.9 × 10^‒2^	0.989	66.73
Mn:ZnS + Sunlight	2.0 × 10^‒2^	0.9951	71.4
Pure ZnS + UV	2.77 × 10^‒2^	0.994	81.8
Mn:ZnS + UV	3.21 × 10^‒2^	0.989	86

## References

[1] Khetan S.K., Collins T.J. (2007). Human pharmaceuticals in the aquatic environment: A challenge to Green Chemistry. Chem. Rev..

[2] Duong H.A., Pham N.H., Nguyen H.T., Hoang T.T., Pham H.V., Pham V.C., Berg M., Giger W., Alder A.C. (2008). Occurrence, fate and antibiotic resistance of fluoroquinolone antibacterials in hospital wastewaters in Hanoi, Vietnam. Chemosphere.

[3] Hu B., Cai F., Chen T. (2015). Hydrothermal synthesis g-C_3_N_4_/Nano-InVO_4_ nanocomposites and enhanced photocatalytic activity for hydrogen production under visible light irradiation. ACS Appl. Mater. Interf..

[4] Trovo A.G., Nigeria R.F., Aguera A., Fernandez-Alba A.R., Malato S. (2011). Degradation of the antibiotic amoxicillin by photo-Fenton process-chemical and toxicological assessment. Water Res..

[5] Li Y., Bi E., Chen H. (2017). Sorption Behavior of Ofloxacin to Kaolinite: Effects of pH, Ionic Strength, and Cu(II). Water Air Soil Pollut..

[6] Kong D., Liang B., Yun H., Cheng H., Ma J., Cui M., Wang A., Ren N. (2015). Cathodic degradation of antibiotics: Characterization and pathway analysis. Water Res..

[7] Alexandrino D.A.M., Mucha A.P., Almeida C.M.R., Gao W., Jia Z., Carvalho M.F. (2017). Biodegradation of the veterinary antibiotics enrofloxacin and ceftiofur and associated microbial community dynamics. Sci. Total Environ..

[8] Zwiener C., Glauner T., Frimmel F.H. (2000). Biodegradation of Pharmaceutical Residues Investigated by SPE-GC/ITD-MS and On-Line Derivatization. J. High Resolut. Chromatogr..

[9] Bellona C., Drewes J.E., Xu P., Amy G. (2004). Factors affecting the rejection of organic solutes during NF/RO treatment--a literature review. Water Res..

[10] Nghiem L.D., Schafer A.I., Elimelech M. (2005). Pharmaceutical retention mechanisms by nanofiltration membranes. Environ. Sci. Technol..

[11] Hartig C., Ernst M., Jekel M. (2001). Membrane filtration of two sulphonamides in tertiary effluents and subsequent adsorption on activated carbon. Water Res..

[12] Westerhoff P., Yoon Y., Snyder S.A., Wert E. (2005). Fate of Endocrine-Disruptor, Pharmaceutical, and Personal Care Product Chemicals during Simulated Drinking Water Treatment Processes. Environ. Sci. Technol..

[13] Zwiener C., Frimmel F.H. (2000). Oxidative treatment of pharmaceuticals in water. Water Res..

[14] Sun J.H., Sun S.P., Wang G.L., Qiao L.P. (2007). Degradation of azo dye Amido black 10B in aqueous solution by Fenton oxidation process. Dyes Pigments.

[15] Sun J.H., Dong S.Y., Wang Y.K., Sun S.P. (2009). Preparation and photocatalytic property of a novel dumb bell-shaped ZnO microcrystal photocatalyst. J. Hazard. Mater..

[16] Ekemena O., Oseghe A., Ofomaja E. (2018). Facile microwave synthesis of pine cone derived C-doped TiO_2_ for the photodegradation of tetracycline hydrochloride under visible- LED light. J. Environ. Manag..

[17] Tobajas M., Belver C., Rodriguez J.J. (2017). Degradation of emerging pollutants in water under solar irradiation using novel TiO_2_-ZnO/clay nanoarchitectures. Chem. Eng. J..

[18] Ponnaiah S.K., Periakaruppan P., Vellaichamy B., Nagulan B. (2018). Efficacious separation of electron-hole pairs in CeO_2_–Al_2_O_3_ nanoparticles embedded GO heterojunction for robust visible-light driven dye degradation. J. Coll. Interf. Sci..

[19] Shamsipur M., Rajabi H.R. (2014). Study of photocatalytic activity of ZnS quantum dots as efficient nanoparticles for effect of ferric ion doping. Spectrochim. Acta Part A.

[20] Shamsipur M., Rajabi H.R., Khani O. (2013). Pure and Fe^3+^-doped ZnS quantum dots as novel and efficient nanophotocatalysts: Synthesis, characterization and use for decolorization of Victoria blue R. Mater. Sci. Semicond. Process..

[21] Rajabi H.R., Farsi M. (2015). Effect of transition metal ion doping on the photocatalytic activity of ZnS quantum dots: Synthesis, characterization, and application for dye decolorization. J. Mol. Catal. A Chem..

[22] Rajabi H.R., Farsi M. (2015). Quantum dot based photocatalytic decolorization as an efficient and green strategy for the removal of anionic dye. Mater. Sci. Semicond. Process..

[23] Rajabi H.R., Karimia F., Kazemdehdashti H., Kavoshi L. (2018). Fast Sonochemically-Assisted Synthesis of Pure and Doped Zinc Sulfide Quantum Dots and their Applicability in Organic Dye Removal from Aqueous Media. J. Photochem. Photobiol..

[24] Pandey V., Tripathi V.K., Singh K.K., Bhatia T., Upadhyay N.K., Goyal B., Pandey G., Hwang I., Tandon P. (2019). Nitrogen donor ligand for capping ZnS quantum dots: A quantum chemical and toxicological insight. RSC Adv..

[25] Chang L., Wu H., He X., Chen L., Zhang Y. (2017). A Highly Sensitive Fluorescent Turn-On Biosensor for Glycoproteins Based on Boronic Acid Functional Polymer Capped Mn-Doped ZnS Quantum Dots. Anal. Chim. Acta.

[26] Sharma S., Dutta V., Singh P., Raizada P., Sani A.R., Bandegharaei A.H., Thakur V.K. (2019). Carbon quantum dot supported semiconductor photocatalysts for efficient degradation of organic pollutants in water: A review. J. Clean. Prod..

[27] Patel J., Jain B., Singh A.K., Susan M.A.B.H., Paul L.J. (2020). Mn-Doped ZnS Quantum dots–An Effective Nanoscale Sensor. Microchem. J..

[28] Abha K., Nebu J., Anjali Devi J.S., Aparna R.S., Anjana R.R., Aswathy A.O., George S. (2019). Photoluminescence Sensing of Bilirubin in Human Serum Using L-Cysteine Tailored Manganese Doped Zinc Sulphide Quantum Dots. Sens. Actuat.-B-Chem..

[29] Wang Y., Yang M., Ren Y., Fan J. (2018). Cu-Mn Codoped ZnS Quantum Dots Based Ratiometric Fluorescent Sensor for Folic Acid. Anal. Chim. Acta.

[30] Chen Z., Li D., Zhang W., Shao Y., Chen T., Sun M., Fu X. (2009). Photocatalytic degradation of dyes by ZnIn_2_S_4_ microspheres under visible light irradiation. J. Phys. Chem. C.

[31] Wang X., Shen S., Jin S., Yang J., Li M., Wang X., Hana H., Li C. (2013). Effects of Zn^2+^and Pb^2+^ dopants on the activity of Ga_2_O_3_-based photocatalysts for watersplitting. Phys. Chem. Chem. Phys..

[32] Norris D.J., Efros A.L., Erwin S.C. (2008). Doped nanocrystals. Science.

[33] Bhargava R.N., Gallagher D., Hong X., Nurmikko A. (1994). Optical properties of manganese-doped nanocrystals of ZnS. Phys. Rev. Lett..

[34] Wu P., Yan X.P. (2013). Doped quantum dots for chemo/biosensing and bioimaging. Chem. Soc. Rev..

[35] Eilers J., Groeneveld E., de Mello Donega C., Meijerink A. (2012). Optical Properties of Mn-Doped ZnTe magic size nanocrystals. J. Phys. Chem. Lett..

[36] Erwin S.C., Zu L., Haftel M.I., Efros A.L., Kennedy T.A., Norris D.J. (2005). Doping semiconductor nanocrystals. Nature.

[37] Yang Y., Chen O., Angerhofer A., Cao Y.C. (2006). Radial-Position-Controlled Doping in CdS/ZnS Core/Shell Nanocrystals. J. Am. Chem. Soc..

[38] Pradhan N., Goorskey D., Thessing J., Peng X.G. (2005). An alternative of CdSe nanocrystal emitters: Pure and tunable impurity emissions in ZnSenanocrystals. J. Am. Chem. Soc..

[39] Biju V. (2014). Chemical modifications and bioconjugate reactions of nanomaterials for sensing, imaging, drug delivery and therapy. Chem. Soc. Rev..

[40] Zhou J., Liu Y., Tang J., Tang W. (2017). Surface ligands engineering of semiconductor quantum dots for chemosensory and biological applications. Mater. Today.

[41] Zhou C., Song J., Zhou L., Zhong L., Liu J., Qi Y. (2015). Greener Synthesis and Optimization of Highly Photoluminescence Mn^2+^-Doped ZnS Quantum Dots. J. Lumin..

[42] Singhal M., Sharma J.K., Jeon H.C., Kang T.W., Kumar S. (2016). Effect of Pyridine Capping on Morphological and Optical Properties of Zns:Mn^2+^ Core–Shell Quantum Dots. J. Mater. Sci. Mater. Electron..

[43] La Porta F.A., Ferrer M.M., Santana Y.V.B., Raubach C.W., Longo V.M., Sambrano J.R., Longo E., Andres J., Li M.S., Varela J.A. (2013). Synthesis of Wurtzite ZnS Nanoparticles Using the Microwave Assisted Solvothermal Method. J. Alloys Compd..

[44] Qadri S.B., Skelton E.F., Hsu D., Dinsmore A.D., Yang J., Gray H.F., Ratna B.R. (1999). Size-induced transition-temperature reduction in nanoparticles of ZnS. Phys. Rev. B.

[45] Rofouei M.K., Tajarrod N., Farahani M.M. (2015). A New Fluorescence Sensor for Cerium (III) Ion Using Glycine Dithiocarbamate Capped Manganese Doped ZnS Quantum Dots. J. Fluoresc..

[46] Geszke-Moritz M., Piotrowska H., Murias M., Balan L., Moritz M., Lulek J., Schneider R.J. (2013). Thioglycerol-capped Mn-doped ZnS quantum dot bioconjugates as efficient two-photon fluorescent nano-probes for bioimaging. Mater. Chem. B.

[47] Kolmykov O., Coulon J., Lalevée J., Alem H., Medjahdi G., Schneider R. (2014). Aqueous Synthesis of Highly luminescent Glutathione-capped Mn^2+^ -doped ZnS Quantum Dots. Mater. Sci. Eng. C.

[48] Karikalan V., Panneerselvam A., Vallalperuman K. (2018). Physico—Chemical Analysis on Cetylpyridinium Chloride (Cpc) with Alcohol Solution at Different Temperatures—Ultrasonic, UV and FTIR Analysis. Dig. J. Nanomater. Bios..

[49] Kung K.H.S., Hayes K.F. (1993). Fourier Transform Infrared Spectroscopic Study of the Adsorption of Cetyltrimethylammonium Bromide and Cetylpyridinium Chloride on Silica. Langmuir.

[50] Sotelo-Gonzalez E., Roces L., Garcia-Granda S., Fernandez-Arguelles M.T., Costa-Fernandez J.M., Sanz-Medel A. (2013). Influence of the Mn^2+^ concentration on Mn^2+^ -doped ZnS Quantum Dots Synthesis: Evaluation of the Structural and Photoluminescent Properties. Nanoscale.

[51] Shah S.I., Li W., Huang C.P., Jung O., Ni C. (2002). Study of Nd^3+^, Pd^2+^, Pt^4+^, and Fe^3+^Dopant Effect on Photoreactivity of TiO_2_ Nanoparticle. PNAS.

[52] Tauc J. (1966). Optical Properties of Solids.

[53] Pouretedal H.R., Keshavarz M.H., Yosefi M.H., Shokrollahi A., Zali A. (2009). Photodegradation of HMX and RDX in the Presence of Nanocatalyst of Zinc Sulfide Doped with Copper. Iran. J. Chem. Chem. Eng..

[54] Pourahmad A. (2012). Photocatalytic Activity of Quantum Dots Incorporated in Molecular Sieves for Generation of Hydrogen. Spectrochim. Acta A.

[55] Kumar R.S., Veeravazhuthi V., Muthukumarasamy N., Thambidurai M., Vishnu Shankar D. (2015). Effect of nickel doping on structural and optical properties of ZnS nanoparticles. Superlattice Microst..

[56] Guo C., Gao S., Lv J., Hou S., Zhang Y., Xu J. (2017). Assessing the photocatalytic transformation of norfloxacin by BiOBr/iron oxides hybrid photocatalyst: Kinetics, intermediates, and influencing factors. Appl. Catal. B-Environ..

[57] Ahmad I., Bano R., Musharraf S.G., Sheraz M.A., Ahmed S., Tahir H., Arfeen Q., Bhatti M.S., Shad Z., Hussain S.F. (2015). Photodegradation of Norfloxacin in aqueous and organic solvents: A kinetic study. J. Photochem. Photobiol. A.

[58] Park H.R., Chung K.Y., Lee H.C., Lee J.K., Bark K.M. (2000). Ionization and Divalent Cation Complexation of Quinolone Antibiotics in Aqueous Solution. Bull. Korean Chem. Soc..

[59] D’Andrea G., Di Nicolantonio G. (2002). A Graphical Approach to Determine the Isoelectric Point and Charge of Small Peptides from pH 0 to 14. J. Chem. Educ..

[60] Mahmoodi N.M., Arami M., Limaee N.Y., Tabrizi N.S. (2006). Kinetics of heterogeneous photocatalytic degradation of reactive dyes in an immobilized TiO_2_ photocatalytic reactor. J. Coll. Interf. Sci..

[61] Lair A., Ferronato C., Chovelon J.M., Herrmann J.M. (2008). Naphthalene degradation in water by heterogeneous photocatalysis: An investigation of the influence of inorganic anions. J. Photochem. Photobiol. A Chem..

[62] Wang C.C., Lee C.K., Lyu M.D., Juang L.C. (2008). Photocatalytic degradation of C.I. Basic Violet 10 using TiO_2_ catalysts supported by Y zeolite: An investigation of the effects operational parameters. Dyes Pigments.

[63] Pouretedal H.R., Norozi A., Keshavarz M.H., Semnani A. (2009). Nanoparticles of zinc sulfide doped with manganese, nickel and copper as nanophotocatalyst in the degradation of organic dyes. J. Hazard. Mater..

[64] Asilturka M., Sayılkana F., Arpac E. (2009). Effect of Fe^3+^ ion doping to TiO_2_ on the photocatalytic degradation of Malachite Green dye under UV and vis-irradiation. J. Photochem. Photobiol. A.

[65] Mahyari M., Bide Y., Gavgani J.N. (2016). Iron(III) porphyrin supported on S and N codoped graphene quantum dot as an efficient photocatalyst for aerobic oxidation of alcohols under visible light irradiation. Appl. Catal. A.

[66] Cong Y., Zhang J., Chen F., Anpo M. (2007). Synthesis and characterization of nitrogen doped TiO_2_ nanophotocatalyst with high visible light activity. J. Phys. Chem. C.

[67] Rajabi H.R., Khani O., Shamsipur M., Vatanpour V. (2013). High-performance pure and Fe^3+^-ion doped ZnS quantum dots as green nanophotocatalysts for the removal of malachite green under UV-light irradiation. J. Hazard. Mater..

[68] Beydoun D., Amal R., Low G., Evoy S.M. (1999). Role of nanoparticles in photocatalysis. J. Nanopart. Res..

[69] Sun L., Liu C., Liao C., Yan C. (1999). ZnS nanoparticles doped with Cu(I) by controlling coordination and precipitation in aqueous solution. J. Mater. Chem..

[70] Montazerozohori M., Nasr-Esfahani M., Joohari S. (2012). Photocatalytic degradation of an organic dye in some aqueous buffer solutions using nano titanium dioxide: A kinetic study. Environ. Prot. Eng..

[71] Kumar K., Chitkara M., Sandhua I.S., Mehta D., Kumar S. (2015). Photocatalytic and magnetic properties of Zn_1-x_Cr_x_O nanocomposites prepared by coprecipitation method. Mater. Sci. Semicond. Proc..

[72] Fujishima A., Zhang X. (2006). Titanium dioxide photocatalysis: Present situation and future approaches. Comptes Rendus Chim..

[73] Barakat M.A., Schaeffer H., Hayes G., Ismat-Shah S. (2004). Photocatalytic degradation of 2-chlorophenol by Co-doped TiO_2_ nanoparticles. Appl. Catal. B Environ..

[74] Kaur A., Kansal S.K. (2016). Bi_2_WO_6_nanocuboids: An efficient visible light active photocatalyst for the degradation of levofloxacin drug in aqueous phase. Chem. Eng. J..

[75] Zhang L.S., Wong K.H., Zhang D.Q., Hu C., Yu J.C., Chan C.Y., Wong P.K. (2009). Zn:In(OH)_*y*_S_*z*_ solid solution nanoplates: Synthesis, characterization, and photocatalytic mechanism. Environ. Sci. Technol..

[76] Guo H., Gao N., Yang Y., Zhang Y. (2016). Kinetics and transformation pathways on oxidation of fluoroquinolones with thermally activated persulfate. Chem. Eng. J..

[77] An T., Yang H., Song W., Li G., Luo H., Cooper W.J. (2010). Mechanistic considerations for the advanced oxidation treatment of fluoroquinolone pharmaceutical compounds using TiO_2_ heterogeneous catalysis. J. Phys. Chem. A.

[78] Chen M., Chu W. (2015). Photocatalytic degradation and decomposition mechanism of fluoroquinolones norfloxacin over bismuth tungstate: Experiment and mathematic model. Appl. Catal. B Environ..

[79] Huang M., Zhou T., Wu X., Mao J. (2017). Distinguishing homogeneous-heterogeneous degradation of norfloxacin in a photochemical Fenton-like system (Fe_3_O_4_/UV/ oxalate) and the interfacial reaction mechanism. Water Res..

[80] Ding D.H., Liu C., Ji Y.F., Yang Q., Chen L.L., Jiang C.L., Cai T.M. (2017). Mechanism insight of degradation of norfloxacin by magnetite nanoparticles activated persulfate: Identification of radicals and degradation pathway. Chem. Eng. J..

[81] Guo H.G., Ke T.L., Gao N.Y., Liu Y., Cheng X. (2017). Enhanced degradation of aqueous norfloxacin and enrofloxacin by UV-activated persulfate: Kinetics, pathways and deactivation. Chem. Eng. J..

[82] Liu C., Nanaboina V., Korshin G.V., Jiang W. (2012). Spectroscopic study of degradation products of ciprofloxacin, norfloxacin and lomefloxacin formed in ozonated wastewater. Water Res..

[83] Zhang X.X., Li R.P., Jia M.K., Wang S.L., Huang Y.P., Chen C.C. (2015). Degradation of ciprofloxacin in aqueous bismuth oxybromide (BiOBr) suspensions under visible light irradiation: A direct hole oxidation pathway. Chem. Eng. J..

[84] Gou J.F., Ma Q.L., Deng X.Y., Cui Y.Q., Zhang H.X., Cheng X.W., Li X.L., Xie M.Z., Cheng Q.F. (2017). Fabrication of Ag_2_O/TiO_2_-Zeolite composite and its enhanced solar light photocatalytic performance and mechanism for degradation of norfloxacin. Chem. Eng. J..

[85] Tang L., Wang J.J., Zeng G.M., Liu Y.N., Deng Y.C., Zhou Y.Y., Tang J., Wang J.J., Guo Z. (2016). Enhanced photocatalytic degradation of norfloxacin in aqueous Bi_2_WO_6_ dispersions containing nonionic surfactant under visible light irradiation. J. Hazard. Mater..

[86] Yang H., Mei L., Wang P., Genereux J., Wang Y., Yi B., Au C., Dang L., Feng P. (2017). Photocatalytic degradation of norfloxacin on different TiO_2-X_ polymorphs under visible light in water. RSC Adv..

[87] Shankaraiah G., Poodari S., Bhagawan D., Himabindu V., Vidyavathi S. (2016). Degradation of antibiotic norfloxacin in aqueous solution using advanced oxidation processes (AOPs)—A comparative study. Desalin. Water Treat..

[88] Nekouei S., Nekouei F. (2018). Photocatalytic degradation of norfloxacin and its intermediate degradation products using nitrogen–doped activated carbon–CuS nanocomposite assisted by visible irradiation. Appl. Organometal. Chem..

[89] Li J., Li R., Zou L., Liu X. (2019). Efficient Degradation of Norfloxacin and Simultaneous Electricity Generation in a Persulfate-Photocatalytic Fuel Cell System. Catalysts.

[90] Zhang G., Xue Y., Wang Q., Wang P., Yao H., Zhang W., Zhao J., Li Y. (2019). Photocatalytic oxidation of norfloxacin by Zn_0.9_Fe_0.1_S supported on Ni foam under visible light irradiation. Chemosphere.

[91] Shah N.S., Khan J.A., Sayed M., Khan Z.H., Rizwan A.D., Muhammad N., Boczkaj G., Murtaza B., Imran M.M., Khan H.M. (2018). Solar light driven degradation of norfloxacin using as-synthesized Bi^3+^ and Fe^2+^ codoped ZnO with the addition of HSO_5_^–^: Toxicities and degradation pathways investigation. Chem. Eng. J..

[92] Jin X., Zhou X., Sun P., Lin S., Cao W., Li Z., Liu W. (2019). Photocatalytic degradation of norfloxacin using N-doped TiO_2_: Optimization, mechanism, identification of intermediates and toxicity Evaluation. Chemosphere.

